# Mepolizumab and dupilumab as a replacement to systemic glucocorticoids for the treatment of Chronic Eosinophilic Pneumonia and Allergic Bronchopulmonary Aspergillosis - Case series, Almoosa specialist hospital

**DOI:** 10.1016/j.rmcr.2021.101520

**Published:** 2021-10-01

**Authors:** Safwat A.M. Eldaabossi, Amgad Awad, Neda'a Anshasi

**Affiliations:** a Consultant Pulmonology, Almoosa Specialist Hospital, Saudi Arabia - Associate Professor of Chest Diseases, Faculty of Medicine, Al Azhar University, Egypt; b Consultant Nephrology, Almoosa Specialist Hospital, Saudi Arabia - Lecturer of Internal Medicine, Faculty of Medicine, Al Azhar University, Egypt; c Resident Internal Medicine, Almoosa Specialist Hospital, Saudi Arabia

**Keywords:** Asthma, Eosinophilic pneumonia, Allergic bronchopulmonary aspergillosis

## Abstract

In this case series, we present four patients who had asthma and blood eosinophilia. Two patients were diagnosed with Chronic Eosinophilic Pneumonia (CEP) and the other two with Allergic Bronchopulmonary Aspergillosis (ABPA). Laboratory findings revealed profound peripheral eosinophilia with abnormal chest radiography (alveolar shadows, segmental atelectasis, and cystic changes). Initial improvement (clinical, laboratory, and radiological) occurred with traditional asthma therapy, including systemic corticosteroids. The patients did not tolerate corticosteroid therapy because of weight gain, uncontrolled diabetes, bone fractures, and psychological adverse effects. Mepolizumab (administered to two CEP cases and one ABPA case) and Dupilumab (administered to one ABPA case) were initiated as steroid-sparing agents, resulting in successful therapy without relapse or adverse effects. Mepolizumab, and Interleukin-5 (IL-5) antagonist, targets diseases mediated by eosinophil activity and proliferation. Dupilumab blocks the Interleukin-4/Interleukin-13 pathway and suppresses Type 2 inflammation, including Immunoglobulin E (IgE). Dupilumab resulted in up to 70% drop in total IgE levels from baseline and reduced eosinophil-mediated lung inflammation, despite the presence of normal or increased blood eosinophil counts.

## 1 Introduction

Eosinophilic lung diseases are rare disorders, characterized by having an increased number of eosinophils within the lungs and/or peripheral blood (defined as eosinophil count of 500 × 10 cells/L. Mild eosinophilia 1500, moderate eosinophilia 1500–5000, severe eosinophilia 5000). Several types of eosinophilic pneumonia exist and can occur in any age group. Eosinophilic pneumonia is classified into different categories depending on whether an identifiable cause is found or not. Such causes include medications, environmental triggers, parasitic infections, Hypereosinophilic syndromes, malignancies, Eosinophilic Granulomatosis with Polyangiitis (EGPA), acute and chronic eosinophilia, and ABPA. Most of these conditions are treated with systemic corticosteroids but it has many side effects [1,2]. Here we report four patients with CEP and ABPA treated successfully with subcutaneous Mepolizumab and Dupilumab.

## 2 Case presentation

### 2.1 Case 1

This is a 56-year-old Saudi Arabian lady, known case of type II diabetes mellitus for 15 years on oral hypoglycemic medications. She is a housewife and a never-smoker. In September 2017, she presented with a dry cough and progressive dyspnea of 3 days’ duration. Dyspnea worsened and became at rest. She denied fever, chest pain, or hemoptysis. She had a history of pneumonia and was admitted and treated with broad-spectrum antibiotics in 2016. She had asthma-like symptoms within the past 4 years with seasonal cough, chest tightness, wheeze, and shortness of breath. She had diffuse pruritus but denied arthralgia or joint swelling. Otherwise, systematic examination was unremarkable.

A general physical examination showed an obese lady (body mass index (BMI): 31 kg/m^2^). Her vital signs were: blood pressure (BP) 140/70 mmHg, pulse (PR) 120 beats/minute, respiratory rate (RR) 32 breaths/minute, temperature of (T) 36.9 °C and oxygen saturation (SpO2) of 85% while breathing ambient air (95% on 4 L of oxygen via nasal cannula). Scratch marks were present all over her body. Cardiac examination: neck veins were not congested; heart sounds were accentuated without murmurs. Chest auscultation showed an overuse of accessory muscles with bilateral equal vesicular breath sounds and bibasilar, early, fine inspiratory crepitation's. No lower limb edema, arthritis nor palpable lymphadenopathy.

**Fig. 1:**
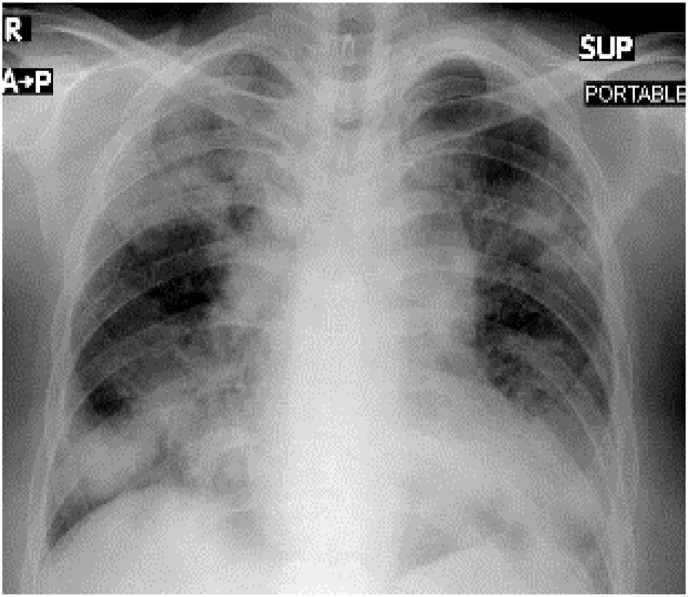
Chest x-ray, posteroanterior (PA) view, January 2019: Bilateral peri-hilar, peri-bronchial cuffing with diffuse, bilateral alveolar shadows more in the right lung.

**Fig. 2:**
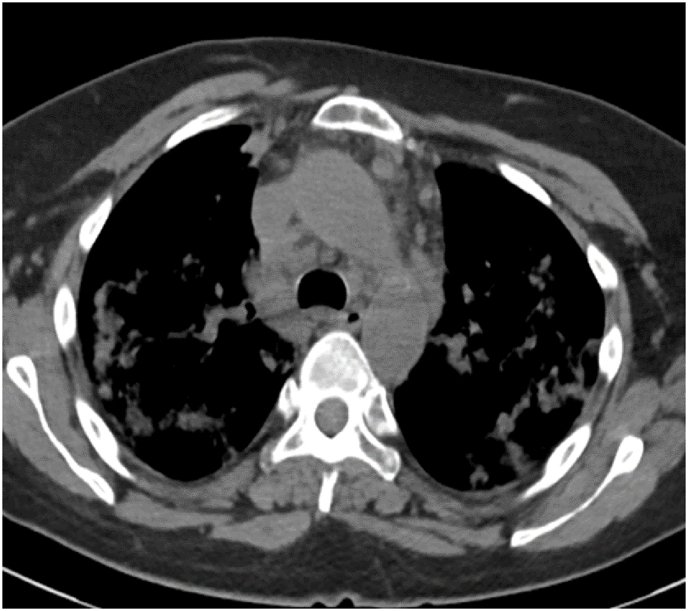
CT chest (mediastinal window), January 2019: there is multiple mediastinal enlarged lymph nodes.

**Fig. 3:**
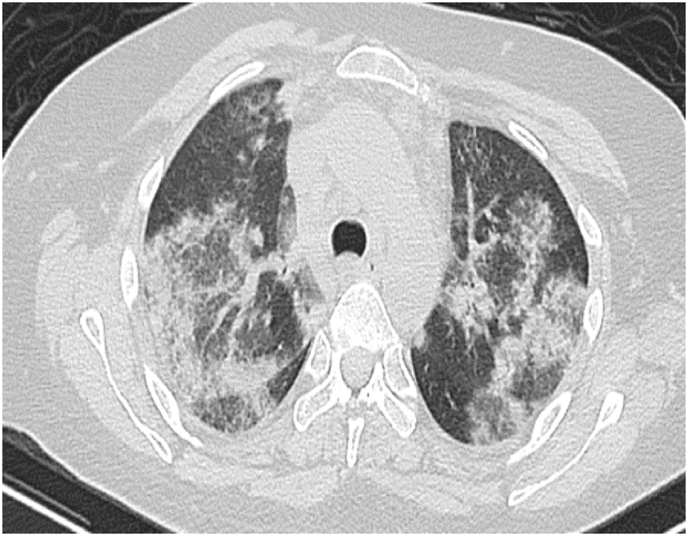
CT chest (lung window), January 2019: Bilateral ground-glass opacities with bilateral bronchial wall thickening of both upper lobes.

Investigation wise, Complete Blood Count (CBC) showed: hemoglobin level of 12.6 g/L, white blood cell (WBC) count of 20.5 × 10^9^/L, and eosinophil count of 59% (12.095 cells/μL). C-reactive protein was 85 mg/L. Her arterial blood gases (ABG) showed partial pressure of oxygen (PaO2) of 52 mmHg and oxygen saturation of 85% on room air. Liver function tests (including prothrombin time) and urine analysis, were unremarkable. Serum creatinine was 125 mmol/L. Chest x-ray showed bilateral consolidations mainly affecting both upper lobes and peripheral zones (Fig. 1). Chest Computed Tomographic (CT) scan revealed bilateral consolidations with multiple enlarged mediastinal lymph nodes (Fig. 2, Fig. 3).

She was initially admitted as a case of community-acquired pneumonia with hypoxemia and empirical antimicrobials were started with oxygen therapy and intravenous fluids. She did not improve and her oxygen requirements were increasing.

The following day, she was admitted to the Intensive Care Unit (ICU) and high doses of intravenous (IV) hydrocortisone was started. She was scheduled for mediastinoscopy for mediastinal lymph node biopsy - to rule out lymphoma, along with flexible bronchoscopy to obtain Bronchoalveolar Lavage (BAL). Abdominal ultrasound showed fatty liver with abdominal lymphadenopathy. Echocardiography was done and was normal apart from mild diastolic dysfunction. Histopathology of the mediastinal lymph node biopsy showed eosinophilic inflammation with the absence of malignant cells. BAL revealed high eosinophilic counts - about 60% of the white blood cell differential, and no pathogens identified on Gram stain, Ziehl-Neelsen stain, and cultures. Further investigations were negative for Antinuclear antibodies (ANAs), and Antineutrophil cytoplasmic antibodies (ANCA). Nerve conduction tests showed no neuropathy.

She was diagnosed as CEP based on the clinical presentation, chest imaging (showing predominantly peripheral or pleural-based, mid-to-upper lung zone opacities), BAL showing eosinophilia (≥25% of the differential), absence of systemic manifestations, negative ANCA, and a normal nerve conduction test. She was managed with high-dose glucocorticoids and discharged 3 days later on oral glucocorticoids which was tapered over 2 months (five-milligram reduction every other day). The trial to wean off oral steroids failed. Her diabetes was uncontrolled and she had 2 bone fractures.

**Fig. 4:**
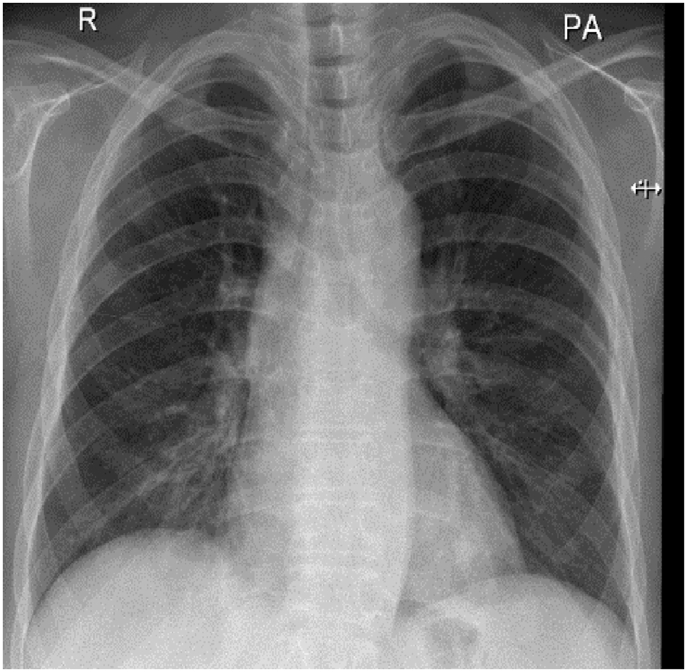
Chest x-ray, PA view, December 2020: normal chest x-ray.

**Fig. 5:**
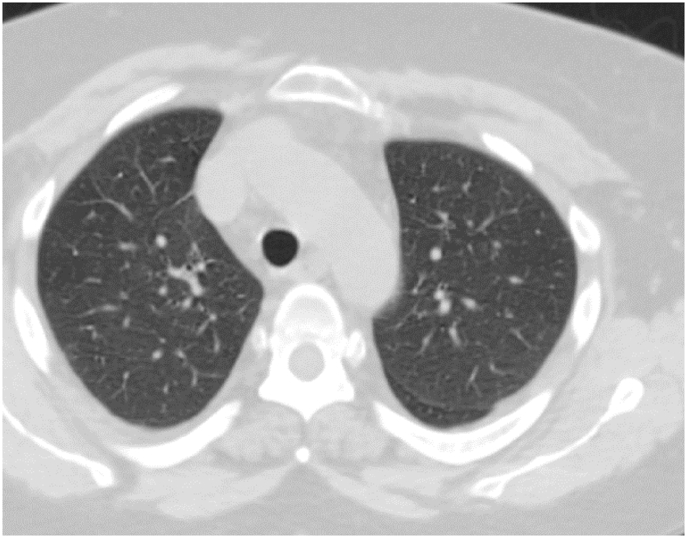
CT chest (lung window), December 2020: normal CT chest.

In December 2019, subcutaneous mepolizumab, 100 mg (mg) monthly, was started and oral corticosteroids were eventually stopped. On regular follow-up, she was asymptomatic till March 2021. Her physical examination was unremarkable. Eosinophil counts dropped to 8%. Chest x-ray and CT chest normalized (Fig. 4, Fig. 5). Her forced expiratory volume in the first second (FEV1) was 55% before therapy and 82% after therapy.

### 2.2 Case 2

Our second case is a 48-year-old Saudi gentleman, a known case of bronchial asthma, allergic rhinitis, and type II diabetes mellitus on metformin. He is a non-smoker. In February 2018, he presented with a dry cough and progressive dyspnea over 5 days. Later on, he developed dyspnea at rest. He was also complaining of nasal congestion and otalgia. He denied fever, chest pain, or hemoptysis nor did he have skin rash, joint pain, or swelling. Other systematic review was unremarkable. He underwent bilateral tymanectomy due to partial deafness. He underwent colonoscopy twice – the last one in 2017, for recurrent abdominal pain and diarrhea and was diagnosed with eosinophilic colitis.

A general physical examination showed an obese man. His vital signs were as follows: BP 140/70 mmHg, PR 120 beats/minute, RR 32 breaths/minute, T 36.9 °C and SpO2 of 89% while breathing ambient air (95% with 2 L’ oxygen via nasal cannula). Cardiac examination: neck veins were not distended with normal, accentuated heart sounds. No audible murmurs. Chest auscultation showed bilateral, equal air entry with vesicular breath sounds, and bibasilar, early fine inspiratory rales. The rest of the physical examination was unremarkable.

**Fig. 6:**
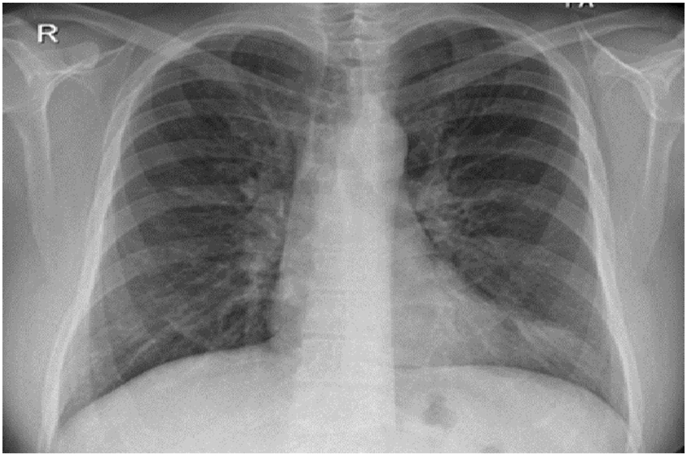
Chest x-ray PA, February 2018: Bilateral, perihilar, peribronchial cuffing with diffuse bilateral alveolar shadows more in the right lung.

**Fig. 7:**
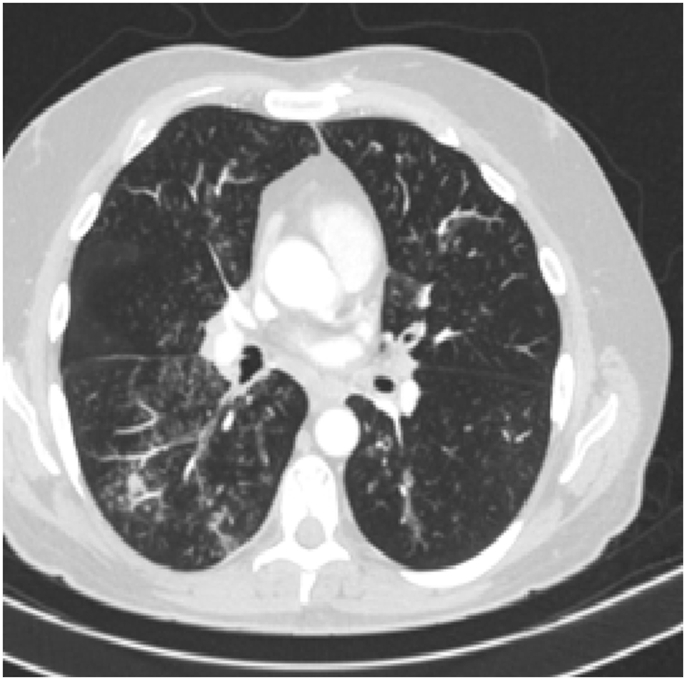
CT chest - lung window, February 2018: bilateral ground-glass opacities with bilateral bronchial wall thickening of both upper lobes and basal segments of the right lower lobe.

**Fig. 8:**
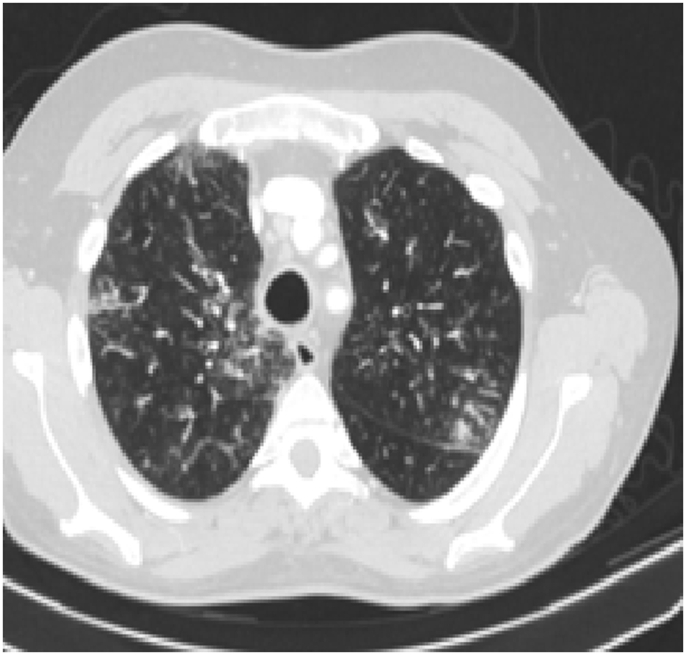
CT chest - lung window, February 2018: bilateral ground-glass opacities with bilateral bronchial wall thickening of both upper lobes and basal segments of the right lower lobe.

His CBC showed hemoglobin of 16.8 g/L, WBC count was 15.6 × 10^9^/L and eosinophils were 42% (6552 cells/μL). C-reactive protein was 80 mg/L. His ABG showed PaO2 of 58 mmHg and oxygen saturation of 87% on room air. Liver function tests and urine analysis were normal. Serum creatinine was 150 mmol/L. His serum Immunoglobulin E (IgE) was 260 IU/Ml and Aspergillus fumigatus Immunoglobulin E (IgE) was negative. Chest x-ray was normal (Fig. 6). CT chest showed multifocal, micronodular, and acino-alveolar opacities primarily raising the possibility of an infectious process likely spread by bronchogenic and hematogenous routes (Fig. 7, Fig. 8).

He was admitted as a case of asthma exacerbation with possible community-acquired pneumonia. Empirical antimicrobials were initiated along with oxygen therapy, intravenous corticosteroids, nebulized bronchodilators, and intravenous fluids. Abdominal CT was suggestive of mesenteric panniculitis with lymphadenopathy in the central, proximal root of the mesentery and also showed right colon pneumatosis coli with few foci of pneumoretroperitoneum. Subsequent investigations were negative for ANAs and ANCA. Nerve conduction tests showed no neuropathy.

He was diagnosed as CEP in a similar fashion as the first case but he didn't undergo BAL. He was treated with high-dose glucocorticoids and discharged 4 days later on oral corticosteroids. The steroid dose was tapered to 5 mg daily over one month. He was receiving inhaled corticosteroid/long-acting β_2_-agonist (ICS/LABA), long-acting muscarinic antagonist (LAMA), and montelukast. The trial to withdraw oral corticosteroids failed and he suffered from weight gain, depression as well as uncontrolled diabetes.

**Fig. 9:**
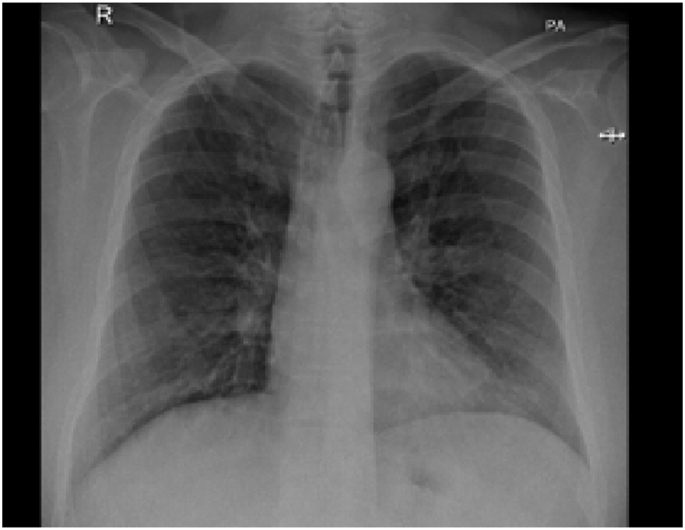
Chest x-ray PA, February 2021: normal.

In September 2020, subcutaneous mepolizumab, 100 mg, monthly was started and the oral corticosteroids were stopped successfully. He remained asymptomatic till his follow-up in March 2021. His examination was unremarkable other than expiratory rhonchi without crackles. Follow-up investigations showed a normal chest x-ray (Fig. 9) and CT chest with a drop in eosinophil count to 5% (270 cells/μL). His current medications are ICS/LABA, LAMA, montelukast, and monthly mepolizumab. His FEV1 was 60% pre-therapy and 72% after therapy.

### 2.3 Case 3

A 31-year-old Saudi male, ex-smoker (quit for 8 months with a smoking index of 10 packs/year), known case of allergic rhinitis, presented in April 2020 with dyspnea of 2 days’ duration associated with left shoulder and axillary pain. He also had intermittent fever. He denied cough and hemoptysis. No other respiratory symptoms. No history of skin rash, joint pain, or swelling. Systematic review was unremarkable.

Physical examination showed: BMI of 24 kg/m^2^, BP 110/70 mmHg, PR 90 beats/minute, RR 20 breaths/minute, T 36.5 °C, and SpO2 of 95% while breathing ambient air. His entire physical examination was unremarkable other than decreased air entry in the left upper chest with dullness to percussion.

**Fig. 10:**
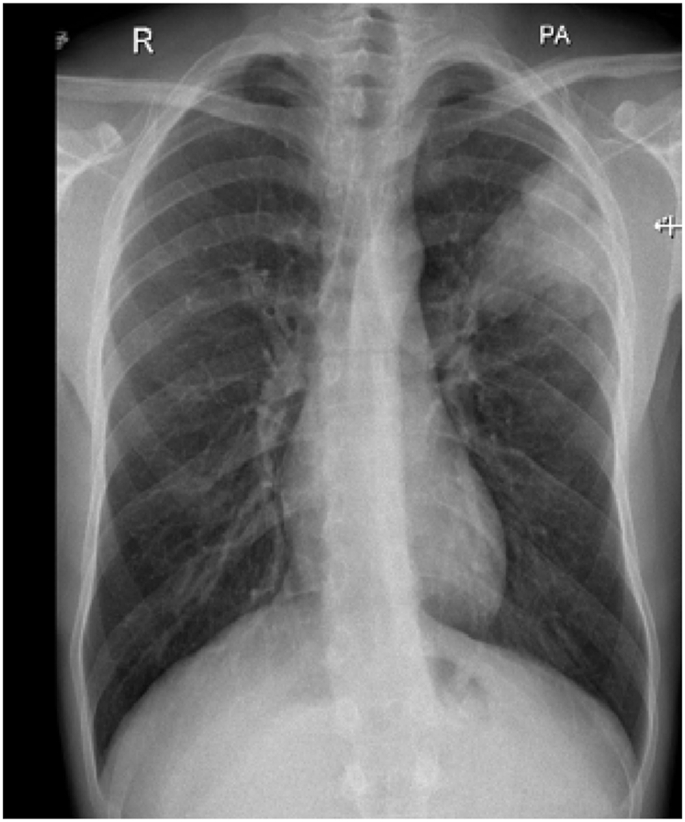
Chest x-ray PA, April 2020: showing left upper lobe mass-like opacity.

**Fig. 11:**
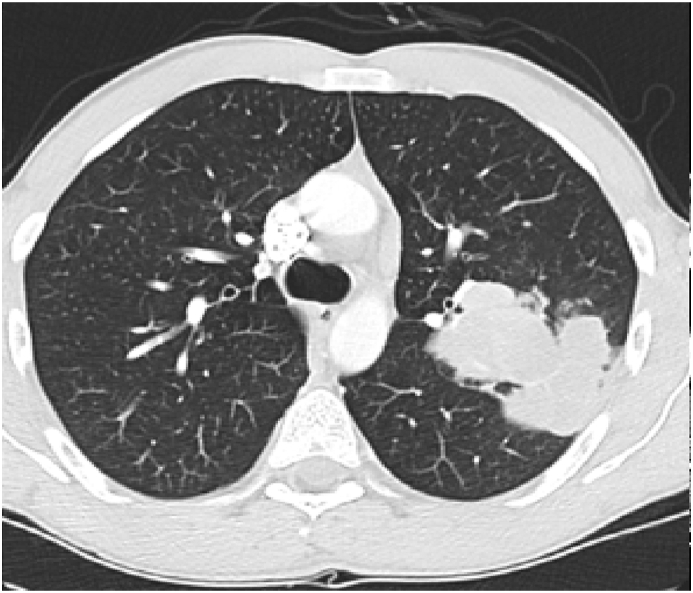
CT chest, April 2020: lung and mediastinal windows respectively, showing a lobulated mass-like lesion.

**Fig. 12:**
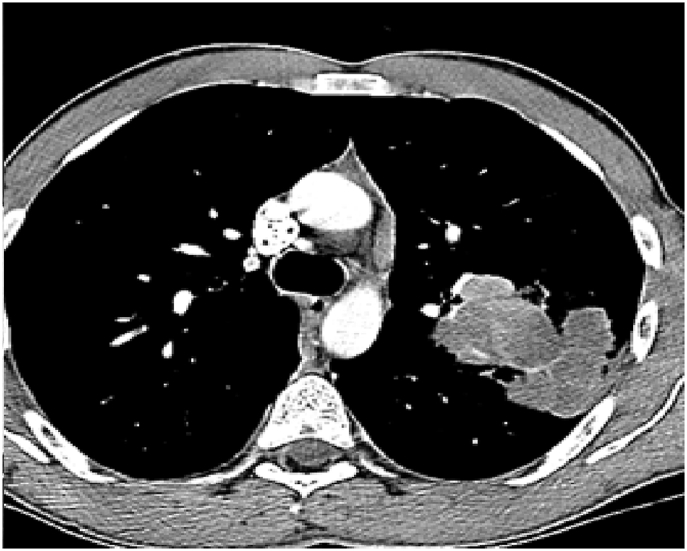
CT chest, April 2020: lung and mediastinal windows respectively, showing a lobulated mass-like lesion.

Investigations: He was swabbed for COVID-19 which was negative. CBC showed: hemoglobin of 14.6 g/L, WBC of 11.40 × 10^9^/L and eosinophil count of 11% (1254 cells/μL). C-reactive protein was 45 mg/L (same labs as previous case). ABG was normal. Liver and renal function tests, as well as urine analysis, were all within normal limits. Chest radiograph showed left upper lobe mass-like lesion (Fig. 10). CT chest showed left upper lobe, lobulated mass with irregular borders (Fig. 11, Fig. 12). Flexible bronchoscopy was done and revealed no endobronchial mass, only viscid secretions obstructing the left apicoposterior segment which was cleared with frequent suctioning. BAL was taken and showed eosinophils of 20%. BAL cultures were negative (bacterial and fungal) as well as galcatomannan test.

On further review of his medical history, he mentions that he had left upper lobe collapse which was treated 2 years ago with flexible bronchoscopy and treatment with antibiotics and corticosteroids. He received antibiotics and oral corticosteroids for three weeks, which resulted in both clinical and radiological improvement.

Two months later he developed a dry cough, dyspnea, chest tightness, and wheezes. His physical examination showed generalized expiratory rhonchi. Spirometry was done and showed a reversible obstructive pattern consistent with bronchial asthma. He was given ICS/LABA and montelukast.

Follow-up investigations showed a WBC count of 11.5 × 10^9^/L, eosinophils of 10%, and his serum Immunoglobulin E (IgE) was more than 10,000 IU/mL. Follow-up chest x-ray and CT chest still showed left upper lobe cystic changes. His Aspergillus fumigatus Immunoglobulin E (IgE) turned out to be positive (class 5). He was diagnosed as ABPA based on a constellation of findings: asthma, elevated serum IgE levels, positive IgE for aspergillus, blood eosinophilia, radiological findings, eosinophilia in BAL, and absence of systemic manifestations.

He was managed with oral steroids and was tapered to 5 mg daily over 1 month. He also received ICS/LABA and montelukast. As with the previous two cases, tapering off steroids was unsuccessful and the patient gained weight and developed anxiety regarding long-term corticosteroid side effects.

**Fig. 13:**
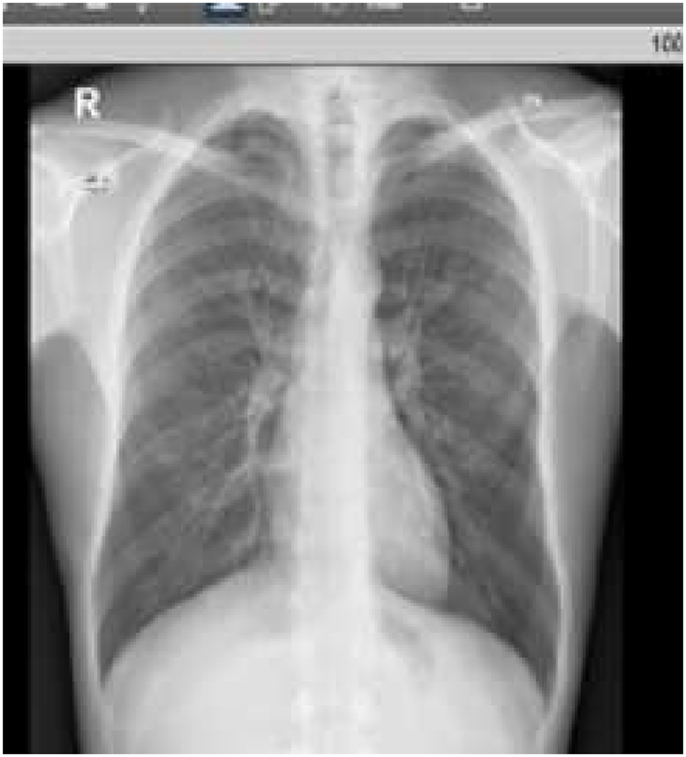
Chest x-ray PA, March 2021: the left upper lobe opacity resolved.

**Fig. 14:**
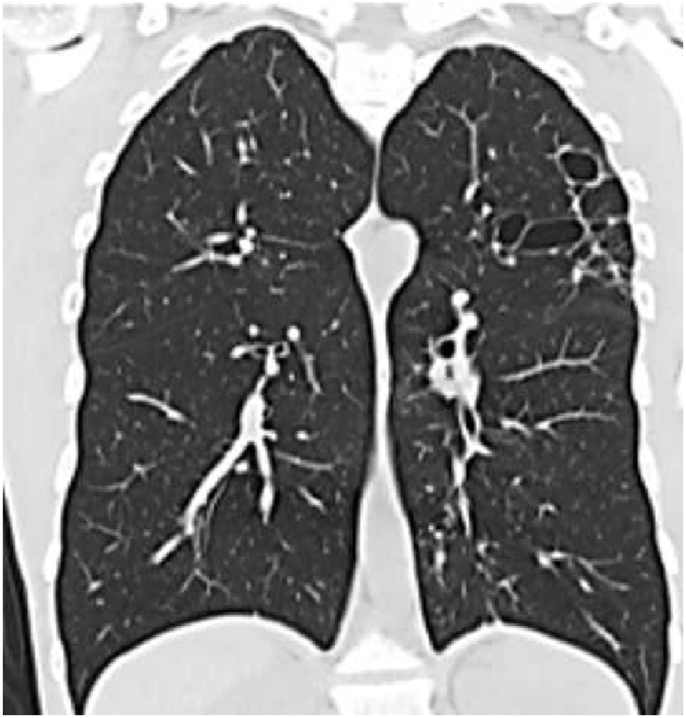
CT chest, March 2021: left upper lobe cystic changes resolved.

In August 2020, subcutaneous mepolizumab, 100 mg monthly was initiated and oral steroids were discontinued. The patient was doing fine on his last follow-up on March 2021. His physical examination was unremarkable. Follow-up investigations showed normal chest x-ray and CT chest (Fig. 13, Fig. 14). He had eosinophils of 4% (230 cells/μL) and an Immunoglobulin E (IgE) level of 289 IU/mL. His current medications are ICS/LABA, montelukast, and monthly mepolizumab. FEV1 was 52% before treatment and 67% post-treatment.

### 2.4 Case 4

Last case is a 25-year-old Saudi gentleman, non-smoker, known case of bronchial asthma (on ICS/LABA and montelukast) and allergic rhinitis (on intranasal steroids) presented in March 2017 with uncontrolled asthma symptoms despite receiving maximal asthma therapy and good compliance to treatment. He was complaining of dry cough, progressive dyspnea, and wheezes that were interfering with his daily activities and sleep. He denied fever, chest pain, and hemoptysis. A review of other systems was unremarkable. He had a history of turbinectomy and nasal polypectomy twice in the past 3 years.

His physical examination showed an obese man with the following vital signs: BP was 130/70 mmHg, PR 105 beats/minute, RR 24 breaths/minute, T 36.9 °C with SpO2 of 95% while breathing ambient air. His Cardiac examination was normal. Chest auscultation revealed bilateral equal air entry with generalized expiratory rhonchi. No skin rashes, arthritis, palpable lymphadenopathy, nor lower limb edema.

**Fig. 15:**
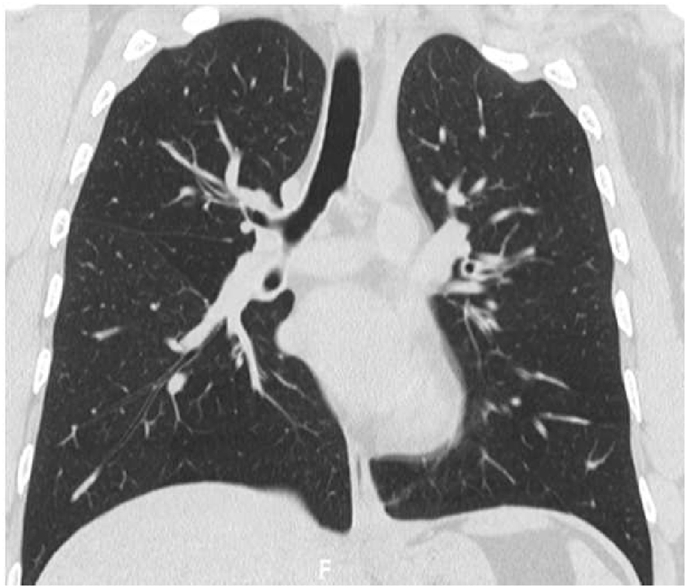
March 2017, CT chest, lung window, showing bilateral bronchial wall thickening and atelectatic bands in the right upper lobe.

Investigations showed: hemoglobin of 16.8 g/L, WBC count of 10.1 × 10^9^/L, eosinophils of 14% (1010 cells/μL), and serum IgE level of 1100 IU/mL. Aspergillus fumigatus IgE was positive (class 5). Liver function tests, renal function tests, and urine analysis, were normal. Chest x-ray was normal as well. CT chest showed bilateral bronchial wall thickening with central bronchiectasis (Fig. 15).

He was diagnosed with ABPA based on the clinical presentation (asthma), laboratory findings (high serum IgE, blood eosinophilia, and positive IgE for aspergillus), and radiological findings. He was on inhaled corticosteroid/long-acting β_2_-agonist/long-acting muscarinic antagonist (ICS/LABA/LAMA), montelukast. Oral corticosteroids were added and gradually tapered to 5 mg daily over one month. This patient also failed to wean off steroids. He developed gastritis with recurrent helicobacter pylori infection and depression.

**Fig. 16:**
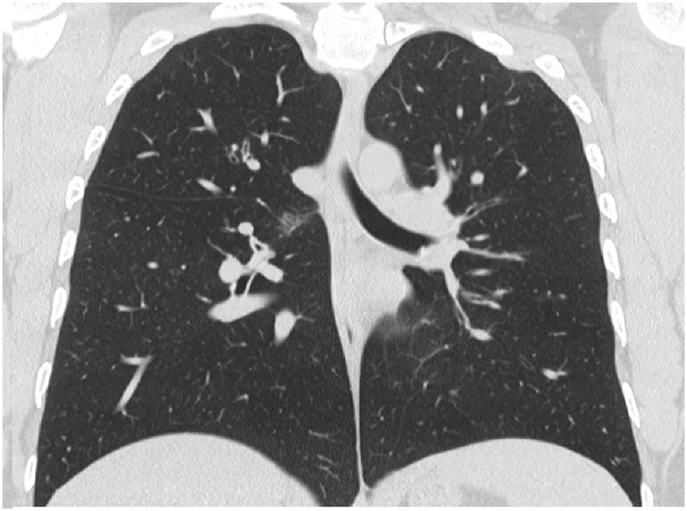
September 2020 CT chest lung window near normal.

In March 2020 the patient received subcutaneous dupilumab 600 mg loading dose followed by 300 mg maintenance dose every 2 weeks. Oral steroids were eventually discontinued. He was stable until March 2021. His physical examination was unremarkable other than generalized expiratory rhonchi. Follow-up investigations showed a normal chest x-ray and CT chest (Fig. 16). His eosinophils increased to 20.2% (2121 cells/μL) and his IgE was 350 IU/mL. His current medications are ICS/LABA/LAMA, montelukast, and dupilumab twice monthly. The FEV1 was 55% pretreatment and 78% post-treatment.

## 3 Discussion

Asthma is less commonly associated with peripheral eosinophilia, however, marked levels of eosinophilia should warrant further investigations [3]. Only three types of pulmonary eosinophilia are associated with bronchial asthma these are CEP, ABPA, and Eosinophilic granulomatosis with polyangiitis (EGPA). If an asthma patient is uncontrolled on ICS/LABA and has a blood eosinophilia (eosinophilic count >500 × 10 cells/L), pulmonary eosinophilia may be considered.

There is limited data on ABPA in Saudi Arabia, probably due to underdiagnoses of the disease or being uncommon in the kingdom [4]. Al-Mobeireek et al. estimated the prevalence of ABPA at 2.7% amongst 264 patients attending an outpatient pulmonary clinic in a tertiary care hospital [5]. The diagnosis of ABPA can be established if four of the following major features are present: history of asthma, positive aspergillus fumigatus diagnostic assay, antibodies to A. fumigatus, elevated total IgE level (417 IU/mL), elevated levels of aspergillus specific IgE, and IgG, peripheral eosinophilia, central bronchiectasis on chest imaging, or pulmonary infiltrates [6]. There is also not much data about the prevalence of CEP in Saudi Arabia - only a couple of cases are reported annually.

Al-Jahdali et al. conducted a retrospective study over a 10-year period and reported 35 patients who met the definition of eosinophilic lung disease. Only 3 cases had ABPA and CEP was not reported [1].

The diagnostic criteria for CEP include respiratory symptoms of at least 2 weeks’ duration, pulmonary and/or peripheral eosinophilia (eosinophil count >25% in BAL/blood eosinophilia ≥1000 cells/μL) and pulmonary infiltrates on chest imaging (the most specific finding is peripheral pulmonary opacities) with the exclusion of other causes of eosinophilia [7].

Asthma and rhinitis are present in 50–60% of the cases and may precede CEP and ABPA by several years. EGPA must be excluded before labeling a patient as CEP. Both ABPA and CEP can present with lung affection and/or rhinitis, however, CEP can present with extrapulmonary manifestations in less than 10% of the cases, such as mononeuritis multiplex, cardiac disorders, colitis, hepatitis, diarrhea, arthralgia, purpura, and urticaria [2].

EGPA commonly affects the skin, musculoskeletal, nervous, and gastrointestinal systems. The diagnosis of EGPA is established when at least 4 of the following features are present: asthma, 10% eosinophils in peripheral blood, sinusitis, pulmonary infiltrates (may be transient), vasculitis (usually small vessels) with extravascular eosinophils on histology, and mononeuritis multiplex or polyneuropathy. Perinuclear antineutrophil cytoplasmic antibody (p-ANCA) can be positive in 50–70% of cases. Medications, malignancies, and parasitic infections must be excluded before making the diagnosis of EGPA [2]. Pulmonary and peripheral eosinophilia can co-exist in eosinophilic lung diseases except in acute eosinophilic pneumonia [8].

The most effective treatment for CEP and ABPA is corticosteroids, which reduce eosinophilia and overall disease activity, prevent pulmonary fibrosis, prevent bronchiectasis, and improves the quality of life. However, relapse is common and patients may require repeated and prolonged courses of corticosteroids which are associated with many adverse effects such as glucose intolerance, hypertension, skin atrophy, osteoporosis, infections, impaired wound healing, and psychiatric disorders [9].

Antifungal agents are available in most countries and are relatively inexpensive compared with biological interventions for ABPA. They improve disease outcomes in both asthma and cystic fibrosis and should be considered in late-stage ABPA or recurrent disease, steroid failure, and/or steroid toxicity [10]. However, reviews have highlighted the weak evidence for the safety and efficacy of azoles [10,11], with only two small, short-term, randomized, double-blind, placebo-controlled trials in asthmatic ABPA and none in cystic fibrosis-ABPA [12,13]. In ABPA, standard medications used for asthma treatment, such as inhaled corticosteroids, long-acting beta agonists, and leukotriene receptor antagonists, are not sufficient to treat symptoms and allergic pneumonia. Systemic corticosteroids are often required. Antifungals are helpful in ABPA but not clearly beneficial in ABPM and SAFS. This then leads to the use of biological modifiers [14]. Antifungal therapy with itraconazole or voriconazole is reserved for patients who are unable to discontinue oral glucocorticoids or have an exacerbation of ABPA [15]. This approach differs from that of the Infectious Diseases Society of America, which recommends itraconazole as part of initial therapy for acute ABPA, with the goal of allowing a reduction in long-term glucocorticoid dose [16].

Biologic therapy with monoclonal antibodies offers targeted therapy and is useful as an alternative to corticosteroids in cases of recurrent disease flares, corticosteroid dependence, or intolerance. Mepolizumab has been shown to reduce the need for corticosteroids in eosinophilic asthma and hyper-eosinophilic syndromes by inhibiting the binding of IL-5 to eosinophils [2,17]. Mepolizumab has also been used effectively in a patient with CEP and a 20-year history of asthma at a dose of 100 mg monthly. However, the duration of therapy with mepolizumab in such patients remains unclear [18]. The use of mepolizumab is off-label in the treatment of CEP but it has induced remission - clinically and radiologically and reduced the use of glucocorticoids [[19], [20], [21]].

In our case series, 2 cases with CEP were treated successfully with mepolizumab resulting in symptom control, weaning of systemic corticosteroids, reducing eosinophil counts, normalization of chest radiography, and improving their lung function tests. In one patient, mepolizumab improved asthma control - instead of using the medications regularly, the patient was using them as needed.

Mepolizumab has already shown clinical benefit in the treatment of sporadic CEP. Otoshi et al. [21], Robert et al. [22], Lawrence and Klings [23], and To et al. [24] also reported successful outcomes in patients with CEP treated with mepolizumab. Allen and Wert conducted an open-label, retrospective study of the treatment of CEP with mepolizumab and found that treatment with mepolizumab was associated with a reduction in disease episodes, steroid use, and improvement in pulmonary infiltrates on imaging [25]. Mepolizumab and dupilumab have been reported to improve symptoms and reduce relapse rates and have a corticosteroid-sparing effect (CEP) [26]. Patients treated for asthma with dupilumab may develop serious systemic eosinophilia, such as eosinophilic pneumonia or vasculitis in the sense of EGPA. A causal relationship between dupilumab and these conditions has not been established [27]. Menzella et al. reported the first case of CEP as a possible side effect of dupilumab used to control severe asthma [27]. Devaraj also reported a case of dupilumab-related eosinophilic pneumonia [28].

We report two cases of severe asthma with ABPA who were successfully treated with mepolizumab (one case) and dupilumab (the other case) after failure of standard asthma therapy (ICS/LABA, LAMA and montelukast) and controlled with a low dose of oral corticosteroids without irreversible fibrosis/bronchiectasis or exacerbation.

In recent years, several studies and case reports have demonstrated the efficacy of anti-IgE antibodies and interleukin-5/interleukin-5 receptor-alpha (IL5/IL5-Rα) antibodies in the treatment of ABPA but was more beneficial for patients with asthma than for patients with CF [[29], [30], [31], [32]]. Mepolizumab has been successfully used to treat patients with ABPA, showing improvement in FEV1, radiographic findings, decrease in total IgE levels, and quality of life without side effects and is considered a glucocorticoid-sparing agent [[29], [33], [34], [35], [36], [37], [38], [39]]. Altman et al. reported that combination therapy with both omalizumab and mepolizumab improved ABPA, and together they may have a potential synergistic effect [31].

Ramonell et al. [38] Muemmler et al. [39] reported that dupilumab therapy for asthma and ABPA resulted in symptom disappearance, a decrease in exacerbations, and complete discontinuation of oral steroids. In 2019, Corren et al. published a post-hoc analysis on the use of dupilumab in 18 patients with asthma and ABPA and reported similar results [40]. ALI et al. [41] and Mikura et al. [42] reported a case of ABPA treated successfully with dupilumab. Two studies also evaluated dupilumab in 21 asthma patients treated for ABPA - IgE levels decreased by 35% from baseline and annual exacerbation rates decreased in 95% of patients [29]. Our patients with ABPA were treated with mepolizumab and dupilumab instead of omalizumab because of the high IgE levels they had.

## 4 Conclusion

This case series demonstrates the utilization of mepolizumab as a steroid-sparing therapy in cases of CEP while dupilumab can be used as a steroid-sparing therapy in ABPA. Mepolizumab and dupilumab therapy could also be beneficial in treating some types of eosinophilic pneumonia owing to their safe side effect profile and low dosing frequency. Further work is needed to confirm the efficacy of these drugs in the treatment of ABPA and CEP.

## Funding declaration

This research did not receive any grants. There was no funding associated with the preparation of this article.

## Declaration of competing interest

The authors have no conflict of interest to declare.
